# Association of breastfeeding and risk of metabolic syndrome and its components in postmenopausal parous women: Korea national health and nutrition examination survey (2010 ~ 2016)

**DOI:** 10.1186/s13690-021-00607-2

**Published:** 2021-05-19

**Authors:** Jusuk Lee, Taehong Kim

**Affiliations:** 1 Department of Pediatrics, Samsung Changwon Hospital, Sungkyunkwan University School of Medicine, Changwon, Republic of Korea; 2 Department of Pediatrics, Pusan National University Yangsan Hospital, 20, Geumo-ro, Mulgeum-eup, Yangsan-si, Gyeongsangnam-do 50612 Republic of Korea

**Keywords:** Breastfeeding, Metabolic syndrome, Postmenopause, Parous women, Korea

## Abstract

**Background:**

Understanding the relationship between breastfeeding (BF) and metabolic syndrome (Mets) is important for maternal long-term health benefits and disease prevention. This study aimed to examine the association between BF and Mets and its components among postmenopausal parous Korean women.

**Methods:**

This cross-sectional study on 10,356 Korean women used nationally representative data from the KNHANES from 2010 to 2016. Anthropometric, laboratory data and manual BP were measured. A multivariate logistic regression analysis was conducted to examine the association of BF with Mets and its components after adjusting for potential confounding variables. A *p*-value < 0.05 was to be considered statistically significant.

**Results:**

Mets was present in 42% of the study participants. The BF group had low household income and education level. The prevalence of Mets in the BF group was higher than that in the non-BF group (42.69% vs. 34.76%, *p* <  0.001). BF was associated with increased risk of Mets (odds ratio [OR]: 1.4, 95% confidence interval [CI]: 1.18–1.65, p <  0.001). The BF group was at higher risks for diabetes (OR: 1.5, 95%CI: 1.14–1.98), hypertension (OR: 1.32, 95%CI: 1.03–1.68), hypertriglyceridemia (OR: 1.42, 95%CI: 1.02–1.99) and low high-density lipoprotein cholesterol (OR: 1.32, 95%CI: 1.06–1.65).

**Conclusion:**

In this study, BF did not affect decreasing the prevalence of Mets and its components.

## Background

Metabolic syndrome (Mets) is a condition characterized by a cluster of cardiovascular risk factors, such as abdominal obesity, insulin resistance/glucose intolerance, dyslipidemia and hypertension, which increases the risk of developing cardiovascular disease and diabetes [1]. The prevalence of Mets is increasing worldwide in recent years [2, 3], and approximately 20–25% of adults have Mets in most countries [4]. The prevalence of Mets in the United States was 34.7% in 2011–2012 (31.0% in men and 36.6% in women) [5]. Li et al. [6] reported that the overall prevalence rate of Mets in China was 24.2% (24.6% in men and 23.8% in women). Meanwhile, 23% of Japanese adults 30 years or older were diagnosed with Mets in 2011 [7]. In Korea, Mets was reported in 26.9% of the entire population (30% in men and 24.6% in women) [8]. Furthermore, women older than 60 years have a six times higher chance of Mets than those 20 to 39 years of age [9].

The benefits of breastfeeding (BF) on infants are well known such as reduced respiratory infections, gastrointestinal tract infections, necrotizing enterocolitis, sudden infant death syndrome, otitis media in children younger than 2 years, allergic disorders (asthma, atopic dermatitis), diabetes, obesity, acute leukemia, hypertension, and neurodevelopmental disorder [10, 11]. BF also has benefits on maternal health such as reduced risk of breast cancer, ovarian cancer, type 2 diabetes [11–13].

However, the effects of BF on Mets are not clearly known. Understanding the relationship between BF and Mets is important for maternal long-term health benefits and disease prevention. In this study, we examined the association between BF and Mets and its components in postmenopausal parous women by analyzing population-based data from the Korea National Health and Nutrition Examination Survey (KNHANES).

## Methods

### Study population

This study was conducted using the raw data of the KNHANES of from 2010 to 2016. The KNHANES is a statutory survey on people’s health behavior, prevalence of chronic diseases, and food and nutrition practices consisting of a cross-sectional survey of a health interview survey, a health examination survey, and a nutrition survey. All survey protocols were approved by the Korea Centers for Disease Control & Prevention. Written informed consent was obtained from all participants before the survey began.

Among the 56,632 participants, 11,396 were postmenopausal parous women. Of these, 1040 subjects were excluded, because 1022 had no information about Mets and 25 subjects did not provide BF data. Finally, 10,356 subjects with complete health interview survey and health examination survey in the final analysis were included.

### Study design

This study employed a cross-sectional design. All data are available from the KNHANES database (http://knhanes.cdc.go.kr/knhanes). Data were categorized into two groups according to breastfeeding status, the BF and non-BF groups. The BF group was determined from the following questionnaires: “Have you been breastfeeding for at least a month?” Medical history and lifestyle data, which included demographic (age, residence), socioeconomic (income level, education level), and lifestyle (use of contraceptives, exercise, smoking, drinking) information were collected using self-reported questionnaires. Education level was divided into three categories: ≤ 9 years (middle school or less), 10 ~ 12 years (high school) and ≥ 13 years (college or more). Income was classified into monthly household income quartiles to determine income level: 1: low, 2: middle low, 3: middle high, 4: high. Regular exercise was indicated as “yes” if the participants performed physical exercise for ≥30 min for at least 3 days per week. Smoking history was divided into three categories based on raw data: current, past, and never a smoker. Alcohol intake was divided into current, past and non-drinking based on monthly drinking rate (when drinking more than once a month for the last 1 year).

### Measurements

All anthropometric measurements were taken by a team of experts by using a consistent, standardized methodology. Height was measured to the nearest 1 mm with a portable extensimeter (SECA225, SECA, Germany). Weight was measured to the nearest 0.1 kg with a calibrated balance scale (GL-6000-20, G-tech, Korea). Body mass index (BMI) was calculated as the ratio of weight to height^2^ (kg/m^2^). Waist circumference (WC) was measured to the nearest 0.1 cm at the narrowest circumference between the lower border of the rib cage and the uppermost borders of the iliac crest, at the end of expiration. Blood pressure (BP) was manually measured three times by well-trained nurses with a mercury sphygmomanometer (Baumanometer Wall Unit 33(0850), Baum, USA) after the participants relaxed for 5 min in a sitting position. Discarded the first measurement and the averages of the second and third measurements as used for analysis.

Blood samples were collected after at least of 8 h (12 h recommended) of fasting, immediately refrigerated after centrifugation, transferred to the Central Testing Institute in Seoul, Korea, and then assayed within 24 h after sampling. Fasting blood glucose, triglycerides, total cholesterol, low-density lipoprotein cholesterol (LDL-C) and high-density lipoprotein cholesterol (HDL-C) level were measured using a Hitachi Automatic Analyzer 7600–210(Hitachi, Japan).

### Definition of metabolic syndrome

The presence of Mets was defined according to the 2009 guideline of the International Diabetes Federation and the American Heart Association / National Heart, Lung, and Blood institute [14]. The criteria for abdominal obesity was defined by the Korean Society for the Study of Obesity [15]. Participants with three or more of the following five components were classified as having Mets: WC ≥85 cm for women, triglycerides ≥150 mg/dL or drug treatment for elevated triglycerides, HDL-C < 50 mg/dL for women or drug treatment for reduced HDL cholesterol, BP (systolic/diastolic BP ≥130/85 mmHg or use of medication for hypertension, and fasting glucose ≥100 mg/dL or under treatment for diabetes.

### Statistical analysis

All statistical analyses were conducted using Statistical Package for the Social Sciences (SPSS) complex sample procedures since KNHANES data were collected through a representative, stratified, and clustered sampling method.

The characteristics of the subjects are presented as estimate and 95% confidence interval (CI). Continuous variables are expressed as mean ± standard error. Chi-square test and independent t-test were applied wherever appropriate. For the assessment of the odds ratios (ORs) of Mets and component of Mets according to BF, multivariate analysis was performed. For the regression analysis, several models were presented, controlling for potentially confounding variables. We first adjusted for age and height (model I), then for the variables in model I plus income, education and residence (model II), and lastly, for variables in model II plus oral contraceptives use, exercise, drinking and smoking (model III). Statistical analysis was conducted using SPSS version 21.0 (IBM Corp., Armonk, NY, US). For all analyses, *p*-values were two-tailed, and *p* < 0.05 was considered statistically significant.

## Results

Table 1 presents the general characteristics of the study participants according to BF. Socioeconomic information (house income, educational level and residence), life style information (oral contraceptives use, regular exercise, drinking and smoking), height, marital status, number of children, age at menarche and age at menopause were statistically significant.

**Table 1:** Characteristics of participants

Variables	Non-breastfeeding (*n* = 905)	Breastfeeding(*n* = 9451)	*P* – value
Age (year) (*n* = 10,356)			< 0.001
< 50s	18.61 (15.36–22.36)	8.17 (7.36–9.05)	
50–59	54.12 (50.31–57.88)	36.69 (35.48–37.91)	
60–69	19.61 (16.93–22.60)	29.12 (28.07–30.20)	
≥ 70	7.66 (6.03–9.69)	26.02 (24.94–27.14)	
Height (cm) (*n* = 10,350)	156.04 ± 0.23	154.19 ± 0.08	< 0.001
Weight (kg) (*n* = 10,349)	57.67 ± 0.39	57.79 ± 0.12	0.761
Marital state (*n* = 10,352)			< 0.001
Single	0.00 (0.00–0.00)	0.00 (0.00–0.12)	
Married	79.74 (76.54–82.60)	70.97 (69.78–72.13)	
Divorced or separated	20.26 (17.40–23.46)	28.99 (27.83–30.18)	
Age at menopause (year) (*n* = 9999)	47.93 ± 0.23	48.96 ± 0.07	< 0.001
Age at menarche (year) (*n* = 10,273)	14.76 ± 0.09	15.53 ± 0.03	< 0.001
Number of children (*n* = 10,356)	3.54 ± 0.07	4.66 ± 0.03	< 0.001
No. breastfed children (*n* = 9451)		2.85 ± 0.02	
Breastfeeding duration (months) (*n* = 9352)		45.57 ± 0.60	
House Income (*n* = 10,280)			< 0.001
1 (low)	14.01 (11.49–16.98)	30.20 (28.92–31.50)	
2 (middle low)	27.75 (24.40–31.36)	26.48 (25.36–27.64)	
3 (middle high)	25.71 (22.40–29.32)	22.64 (21.57–23.75)	
4 (high)	32.53 (28.84–36.44)	20.68 (19.52–21.88)	
Education level (*n* = 10,339)			< 0.001
≤ 9 years	36.43 (32.74–40.28)	69.56 (68.10–70.98)	
10 ~ 12 years	39.99 (36.23–43.87)	21.27 (20.16–22.42)	
≥ 13 years	23.58 (20.15–27.40)	9.17 (8.33–10.07)	
Occupation (*n* = 10,347)			0.246
No	53.80 (49.72–57.83)	56.31 (54.90–57.70)	
Yes	46.20 (42.17–50.28)	43.69 (42.30–45.10)	
Residence (*n* = 10,356)			< 0.001
Urban	85.52 (82.06–88.42)	75.83 (73.11–78.36)	
Rural	14.48 (11.58–17.94)	24.17 (21.64–26.89)	
Oral Contraceptives use (*n* = 10,346)			< 0.001
No	86.76 (83.90–89.18)	77.74 (76.73–78.71)	
Yes	13.24 (10.82–16.10)	22.26 (21.29–23.27)	
Regular exercise (*n* = 10,345)			0.014
No	79.15 (75.69–82.23)	83.10 (82.01–84.14)	
Yes	20.85 (17.77–24.31)	16.90 (15.86–17.99)	
Drinking status (*n* = 10,289)			< 0.001
No	40.38 (36.76–44.11)	48.88 (47.54–50.23)	
Past	25.76 (22.39–29.44)	23.17 (22.10–24.28)	
Yes	33.86 (30.08–37.85)	27.94 (26.78–29.13)	
Smoking status (*n* = 10,297)			< 0.001
No	87.65 (84.65–90.13)	92.33 (91.58–93.03)	
Past	4.35 (3.03–6.21)	3.50 (3.05–4.02)	
Yes	8.00 (5.90–10.77)	4.17 (3.66–4.74)	

Estimate (95% confidence interval) for categorical variables and mean with standard error for continuous variables

Mets was present in 42% of the study participants. The prevalence of Mets in the BF group was higher than that in the non-BF group. Regarding the components of Mets, the BF group had significantly higher prevalence of abdominal obesity, hypertension, hypo-HDL-cholesterolemia and higher BMI (Table 2).

**Table 2:** Metabolic syndrome of participants

Variable	Non-breastfeeding (*n* = 905)	Breastfeeding(n = 9451)	*P*-value
Mets (*n* = 10,356)			< 0.001
No	65.24 (61.55–68.75)	57.31 (56.05–58.56)	
Yes	34.76 (31.25–38.45)	42.69 (41.44–43.95)	
WC (≥85 cm) (*n* = 10,356)			< 0.001
No	74.48 (70.81–77.84)	63.53 (62.22–64.82)	
Yes	25.52 (22.16–29.19)	36.47 (35.18–37.78)	
FBS (≥100 mg/dl) or diabetes medication (*n* = 10,356)			0.494
No	62.25 (58.48–65.88)	60.92 (59.70–62.13)	
Yes	37.75 (34.12–41.52)	39.08 (37.87–40.30)	
HBP(≥130/85 mmHg) or antihypertensive medication (*n* = 10,356)			< 0.001
No	55.88 (52.04–59.66)	41.66 (40.35–42.98)	
Yes	44.12 (40.34–47.96)	58.34 (57.02–59.65)	
TG(≥150 mg/dl) or drug treatment for elevated TG (*n* = 10,356)			0.715
No	67.98 (64.23–71.52)	68.70 (67.53–69.84)	
Yes	32.02 (28.48–35.77)	31.30 (30.16–32.47)	
HDL(≤50 mg/dl) or drug treatment for reduced HDL (*n* = 10,356)			0.018
No	46.24 (42.64–49.88)	41.62 (40.42–42.83)	
Yes	53.76 (50.12–57.36)	58.38 (57.17–59.58)	
BMI (≥25 kg/m^2^) (*n* = 10,356)			< 0.001
No	71.26 (67.56–74.69)	62.36 (61.17–63.54)	
Yes	28.74 (25.31–32.44)	37.64 (36.46–38.83)	

Values are presented as estimate (95% confidence interval)

*Abbreviations*: *Mets* Metabolic syndrome, *WC* Waist circumference, *FBS* Fasting blood sugar, *HBP* High blood pressure, *TG* Triglyceride, *HDL* High-density lipoprotein, *BMI* Body mass index

The mean values for the Mets components according to BF are shown in Table 3. The average number of the five Mets components was higher in the BF group than that in the non-BF group. For each of the Mets components, the BF group had significantly higher WC, and systolic and diastolic BP and lower total cholesterol and HDL-C level.

**Table 3:** Mean and standard error value of components of metabolic syndrome

Variable	Non-breastfeeding (*n* = 905)	Breastfeeding (*n* = 9451)	*P*-value
No. of Mets components	1.93 ± 0.05	2.24 ± 0.02	< 0.001
WC (cm)	79.69 ± 0.38	82.21 ± 0.14	< 0.001
FBS (mg/dl)	100.60 ± 0.81	101.83 ± 0.31	0.139
Total cholesterol (mg/dl)	203.35 ± 1.54	199.55 ± 0.45	0.017
TG (mg/dl)	137.87 ± 4.52	134.00 ± 1.02	0.405
HDL (mg/dl)	52.79 ± 0.46	51.08 ± 0.16	< 0.001
LDL (mg/dl)	122.96 ± 2.61	121.11 ± 0.76	0.495
Systolic BP (mmHg)	122.17 ± 0.75	126.33 ± 0.27	< 0.001
Diastolic BP (mmHg)	76.91 ± 0.44	75.79 ± 0.14	0.014

Values are presented as Mean ± standard error

*Abbreviations*: *Mets* Metabolic syndrome, *WC* Waist circumference, *FBS* Fasting blood sugar, *TG* Triglyceride, *HDL* High-density lipoprotein, *LDL* Low-density lipoprotein, *BP* Blood pressure

To determine the association between BF and Mets in all subjects, logistic regression analysis was performed (Table 4). BF was associated with increased risk of Mets in the crude model. The risk of Mets was not statistically significant in model I (after adjustment for age and height), model II (adjusted for age, height, income, education and residence), or model III (adjusted for model II plus oral contraceptive use, exercise, drinking and smoking).

**Table 4:** Logistic regression model of breastfeeding for metabolic syndrome

	Non-breastfeeding	Breastfeeding (Odds ratio(95%CI))	***P***-value
**Crude**	Reference	1.40 (1.18–1.65)	< 0.001
**Model I**	Reference	0.97 (0.81–1.16)	0.737
**Model II**	Reference	0.84 (0.70–1.01)	0.064
**Model III**	Reference	0.84 (0.72–1.01)	0.063

Model I; adjusted for age, height

Model II: adjusted for age, height, income, education, residence

Model III: adjusted for age, height, income, education, residence and use of oral contraceptives, exercise, drinking, smoking

Table 5 shows the ORs for the association of BF and Mets components. Compared with the non-BF group, the BF group was at higher risks for diabetes, hypertension, hypertriglyceridemia, and low HDL-C. After controlling for confounding factors, there was not statistically significant risk.

**Table 5:** Logistic regression model of each component of metabolic syndrome according to breastfeeding

	Non-Breastfeeding	Breastfeeding (OR(95%CI))	***P***-value
**Crude**			
**WC (≥85 cm)**	1	1.14 (0.78–1.67)	0.506
**High FBS (≥100 mg/dl)**	1	1.50 (1.14–1.98)	0.004
**High BP(≥130/85 mmHg)**	1	1.32 (1.03–1.68)	0.029
**Low HDL (≤50 mg/dl)**	1	1.32 (1.06–1.65)	0.013
**High TG (≥150 mg/dl)**	1	1.42 (1.02–1.99)	0.039
**Model I**			
**WC (≥85 cm)**	1	0.79 (0.52–1.20)	0.271
**High FBS (≥100 mg/dl)**	1	1.11 (0.83–1.50)	0.469
**High BP(≥130/85 mmHg)**	1	1.10 (0.85–1.42)	0.469
**Low HDL (≤50 mg/dl)**	1	0.91 (0.72–1.16)	0.451
**High TG (≥150 mg/dl)**	1	0.84 (0.59–1.19)	0.321
**Model II**			
**WC (≥85 cm)**	1	0.76 (0.50–1.16)	0.209
**High FBS (≥100 mg/dl)**	1	1.01 (0.75–1.36)	0.931
**High BP(≥130/85 mmHg)**	1	0.99 (0.76–1.28)	0.921
**Low HDL (≤50 mg/dl)**	1	0.81 (0.64–1.03)	0.079
**High TG (≥150 mg/dl)**	1	0.74 (0.51–1.06)	0.095
**Model III**			
**WC (≥85 cm)**	1	0.76 (0.49–1.17)	0.216
**High FBS (≥100 mg/dl)**	1	1.03 (0.76–1.39)	0.849
**High BP(≥130/85 mmHg)**	1	1.03 (0.79–1.34)	0.837
**Low HDL (≤50 mg/dl)**	1	0.80 (0.63–1.02)	0.070
**High TG (≥150 mg/dl)**	1	0.72 (0.50–1.04)	0.081

1. Model I: adjusted for age, height

2. Model II: adjusted for age, height, income, education, residence

3. Model III: adjusted for age, height, income, education, residence and oral contraceptives, exercise, drinking, smoking

*Abbreviations*: *WC* Waist circumference, *FBS* Fasting blood sugar, *BP* Blood pressure, *HDL* High-density lipoprotein, *TG* Triglyceride

## Discussion

In this population-based cross-sectional study, we investigated the association between BF and Mets and its components among Korean postmenopausal parous women using data from the KNHANES. There are some key findings in this study. First, the BF group had significantly higher prevalence of Mets. Second, the BF group had higher WC and BP and lower total cholesterol and HDL-C levels. Third, the risk of Mets was higher in the BF groups. Among the Mets components, the risk was higher for diabetes, hypertension and dyslipidemia. The results showed that BF does not affect reducing the risks of Mets and its components.

Multiple studies have examined the association between BF and the risk of Mets in postmenopausal women. Several studies found that BF appears to have a protective effect against the development of hypertension [16–19]. Zhang et al. [17] reported that both BF history and duration were associated with reduced risk of hypertension in middle-aged and older mothers. Furthermore, Schwarz et al. [19] reported that the beneficial effect of BF on hypertension seems to persists after menopause, as American women ≥50 years who reported a BF history of more than 12 months had reduced risk of hypertension. Unexpectedly, we found that BF was not associated with a reduced risk of developing hypertension. Similarly, Natland et al. [16] found that mothers aged < 50 years who had never breastfed had higher odds of hypertension than those who had breastfed ≥24 months, but there were no clear associations in mothers ≥50 years. Several studies have shown that the effect reducing the risk of hypertension does not appear to persist into older age [18, 20]. Further studies are needed to better clarify the association between BF and hypertension.

In our study, BF group had lower total cholesterol and HDL-C level. Compared with the non-BF group, the BF group was at higher risk for hypertriglyceridemia and low HDL-C. However, the evidence of the long-term effect of BF on the lipid profile remains inconsistent. Schwarz et al. [19] reported that a long duration of BF resulted in reduced prevalence of hyperlipidemia. Similarly, a study reported a significant inverse association between duration of BF and triglyceride, total cholesterol and LDL-C level [16]. However, the Coronary Artery Risk Development in Young Adults study [21] did not find a significant association of BF with total cholesterol, LDL-C or triglyceride concentration, but it showed a difference in HDL-C between women who breastfed for 3 months or more and women who breastfed for less than 3 months. Other studies reported that BF lowers total cholesterol and triglyceride, but HDL-C level was not related [22, 23].

The incidence of Mets is known to be influenced by various socio-economic factors such as education level, income level, wealth and type of job. Several studies that analyzed the association of education, income level and the prevalence of Mets, showed that the lower the socioeconomic level of women, the higher the prevalence of Mets [24–26]. In addition, the lower socio-economic levels lead to increased insulin resistance and abdominal obesity due to poor lifestyle, nutritional imbalance, stress, etc., resulting in health problems such as Mets and cardiovascular disease [27]. The lower the income levels tend to consume low-cost, high-calorie foods that contain more such things as fat, added sugar, and refined grains [28]. Women with lower education tend to give birth to more children, and these pregnancies and childbirth had been reported to cause metabolic disorders accompanied by increased abdominal obesity, decreased HDL-C, and psychological depression during postpartum and child rearing [29]. In the BF group, many of the subjects had lower household income and education level than non-BF group. Thus, the BF group had higher prevalence of central obesity, hypertension and dyslipidemia and higher risks of diabetes, hypertension and dyslipidemia than the non-BF group. Our results showed that social and environmental factors are more important than BF in the prevalence of Mets.

This study has some limitations. First, the cross-sectional design of the KNHANES made it difficult to conclude a causal relationship between BF and the prevalence of Mets components. Second, self-reporting of BF duration, smoking, drinking and exercise pattern recall may lead to misclassification and measurement error. Furthermore, because it was a survey study, it was not possible to analyze the factors affecting Mets according to the duration of BF, lifestyle and dietary pattern. No information was available about the pregnancy state, such as gestational weight gain or postpartum weight retention, in the current study. Despite these limitations, our study has some strengths. First, we analyzed a nationally representative large sample from the KNHANES. Second, this data were adjusted for confounding factors in our models. These results are especially helpful in establishing evidence-based health policies on women’s health.

## Conclusion

In this population-based study of postmenopausal parous Korean women, BF did not affect decreasing prevalence of Mets or its components. It also showed that social and environmental factors are more important than BF in the prevalence of Mets. Lifestyle changes, such as weight reduction, regular exercise and healthy dietary habits should be emphasized.

## Data Availability

The datasets generated and /or analyzed during the current study are available from the Korea National Health and Nutrition Examination Survey (http://knhanes.cdc.go.kr/knhanes).

## Abbreviations

Mets: Metabolic syndrome
BF: Breastfeeding
KNHANES: Korea National Health and Nutrition Examination Survey
WC: Waist circumference
BMI: Body mass index
BP: Blood pressure
HDL-C: High density lipoprotein - cholesterol
LDL-C: Low density lipoprotein-cholesterol
CI: Confidence interval
OR: Odds ratio
